# *Staphylococcus aureus*-Derived Extracellular Vesicles Enhance the Efficacy of Endocrine Therapy in Breast Cancer Cells

**DOI:** 10.3390/jcm11072030

**Published:** 2022-04-05

**Authors:** Jeongshin An, Hyungju Kwon, Woosung Lim, Byung-In Moon

**Affiliations:** 1 Department of Surgery, Ewha Womans University Mokdong Hospital, School of Medicine, Ewha Womans University, 1071 Anyangcheon-ro, Yangcheon-gu, Seoul 07985, Korea; jsan@ewha.ac.kr (J.A.); hkwon@ewha.ac.kr (H.K.); limw@ewha.ac.kr (W.L.); 2 Institute of Convergence Medicine Research, Ewha Womans University Mokdong Hospital, School of Medicine, Ewha Womans University, 1071 Anyangcheon-ro, Yangcheon-gu, Seoul 07985, Korea

**Keywords:** breast cancer, microbiome, bacterial extracellular vesicle, *Staphylococcus*

## Abstract

The microbiome involved in the human estrogen metabolism is known as the estrobolome. This study aimed to show that the estrobolome can be used in breast cancer treatment. We first analyzed the blood microbiome composition of healthy controls and patients with breast cancer. In particular, we investigated the bacteria producing β−glucuronidase and/or β−galactosidase, which are involved in estrogen metabolism in the human body. *Staphylococcus* species were more abundant in healthy controls than in breast cancer patients and therefore were selected for further analyses. The effect of *Staphylococcus aureus* on endocrine therapy was analyzed by a combination treatment with tamoxifen. Analysis of the microbiome of blood samples showed that species producing β−glucuronidase were more abundant in breast cancer patients than in healthy controls. Further experiments confirmed that the efficacy of tamoxifen increased when administered in conjugation with the extracellular vesicles (EVs) of *S. aureus*. Based on our results, we deduced that *S. aureus* EVs could potentially be used as adjuvants for breast cancer treatment in the future.

## 1. Introduction

The microbiome refers to the genetic material of all microbes in the human body [1]. The human microbiome differs from person to person according to the environment and/or underlying diseases [2]. Although microbiome exhibits individual characteristics, they have common patterns for each disease, including cancer. According to a previous study, blood microbiome analysis can be used to diagnose 33 types of cancers, including breast cancer [3]. This indicates that bacterial 16S rRNA appears to reflect systemic microbiome composition and can be used as a biomarker for disease, as its variation can be used to track the onset and progression of multiple pathologies [4]. Each symbiotic bacterium in the microbiota produces different metabolites that can circulate systemically in the human body [5]. It can affect overall health by affecting processes, including carcinogenesis and cancer protection [6]. Since mammalian EVs are potent regulators of both the innate and adaptive immune systems, these are implicated in the development or progression of cancer [7]. Similarly, bacterial EVs may participate in the prognosis and treatment of neoplasia. In fact, half of the metabolites in blood are produced by symbiotic bacteria in the human body [8], and estrogen metabolism is connected to the estrobolome [9]. In other words, the prognosis of breast cancer after endocrine therapy may differ for each individual due to the presence of different metabolites.

The estrogen−metabolizing microbiome is known as the estrobolome [10]. It regulates the levels of estrogen circulating in the human body [11] and is related to the development and progression of various carcinomas, including breast cancer [12]. More than 70% of all breast cancers are hormone receptor−positive breast cancers [13], and elevated estrogen levels are a risk factor for breast cancer development [14]. Thus, it can be inferred that the estrobolome affects the onset and progression of breast cancer. The estrobolome consists of some bacterial genes encoding β−glucuronidase and/or β−galactosidase that are involved in regulating estrogen metabolism in the human body [15,16]. β−-galactosidase, a homologue of β−glucuronidase, breaks down sugars similar to β−glucuronidase [10]. The bacterial genera producing β−glucuronidase and/or β−galactosidase were compared and analyzed between breast cancer patients and healthy controls. The differences in the estrobolomes of these two groups showed the effect of this microbiome on breast cancer.

*Staphylococcus* sp. producing β−galactosidase are abundant in microbiome of healthy subjects compared to that of patients with breast cancer in this experiment. We chose *S. aureus* for the endocrine experiment. *S. aureus*, which colonizes the nares, skin, and gastrointestinal tract and is a Gram−positive commensal bacterium and an opportunistic pathogen [17]. In particular, according to previous results, Eap protein derived from *S. aureus* prevents adhesion of breast cancer cells and bone metastasis in breast cancer [18]. *S. aureus* may have possibility to decrease the risk of breast cancer even though a relatively low proportion exists in the microbiome of healthy controls. This is the reason why we selected *S. aureus* as one of the microbiome helpful in the treatment of breast cancer in this study. Additionally, the extracellular vesicles (EVs) of *S. aureus* are not infectious but have bacterial characteristics and are being studied for their potential use in therapeutics, for example, as a vaccine platform [19].

Bacterial EVs originate from human symbiotic bacteria and circulate throughout the human body via blood [20]. Approximately 99% of the microbial mass originates from the gastrointestinal tract [6]. Symbiotic bacteria living in the large intestine can be involved in the development of colitis and colorectal cancer due to the cytotoxicity and inflammation caused by their microbial metabolites [21]. However, breast cancer, the carcinoma of organs distant from the gastrointestinal tract, is believed to be partially the result of indirect effects of the microbiome on metabolism [22]. Bacterial EVs exist within body fluids, including blood, carrying metabolites and nucleic acids [23,24]. These EVs in the blood can initiate intracellular signaling cascades via receptors on the host cell surface, triggering research on the use of bacterial EVs for next−generation cancer treatment [25,26]. The present study compared the characteristics of the EVs of the blood microbiota of breast cancer patients to those of a healthy control group to identify a target microbiome and revealed that *S. aureus* EVs have potential applications for treatment.

The anticancer effects of *Staphylococci* have been investigated in previous studies [27,28]. However, to the best of our knowledge, the effect of *S. aureus* EVs on endocrine therapy in breast cancer has not been reported yet. The present study proposes a novel mechanism by which EVs of *S. aureus* within the estrobolome could contribute to breast cancer treatment.

## 2. Materials and Methods

### 2.1. DNA Extraction from Blood Samples

Among the 288 Korean female applicants recruited from the Ewha Womans University Mokdong Hospital and the Inje University Haeundae Hospital to participate in the present study, 192 were healthy controls, and 96 were diagnosed with stage 0–III breast cancer. Male patients and patients who had used antibiotics within a month before collecting samples were excluded, as antibiotics could affect the results of the microbiome analysis. Sera from patients with tumors were collected before surgery or other treatments. This study was approved by the Institutional Review Boards of the Ewha Womans University Mockdong Hospital (IRB No. EUMC 2014-10-005-019) and Inje University Haeundae Hospital (IRB No. 1297992-2015-064).

Blood samples were collected in vacutainer serum separator tubes. EVs were isolated from blood samples using an ultracentrifugation method. Briefly, the collected serum was centrifuged at 3000 rpm for 15 min at 4 °C and mixed with 1× phosphate−buffered saline (PBS, pH 7.4, ML008-01; Welgene, Gyeongsan, Korea). The resulting supernatant was separated, centrifuged at 10,000× *g* for 1 min at 4 °C, and filtered using a 0.22 μm filter. The filtered solution has performed the ultracentrifugation at 150,000× *g* for 3 h at 4 °C on a type 45 Ti rotor (Beckman Instruments, Brea, CA, USA). After ultracentrifugation, the EV pellets were obtained and resuspended in phosphate-buffered saline (PBS). The obtained EVs were stored at −80 °C until use [29]. The DNA from the EVs was extracted using a DNA isolation kit (MoBio PowerSoil DNA Isolation Kit, Qiagen, Hilden, Germany following the manufacturer’s instructions. The extracted DNA was quantified using the QIAxpert system (QIAGEN, Hilden, Germany).

### 2.2. Microbiome Analysis of Blood Samples

Next-generation sequencing was performed using the V3–V4 hypervariable region of the 16S ribosomal DNA (rDNA). The primers used for microbiome analysis were as follows: 16S_V3_F (5′-TCGTCGGCAGCGTCAGATGTGTATAAGAGACAGCCTACGGGNGGCWGCAG-3′) and 16S_V4_R (5′-GTCTCGTGGGCTCGGAGATGTGTATAAGAGACAGGAC-TACHVGGGTATCTAATCC-3′) [30]. Libraries were prepared and used according to the MiSeq System Guide (Illumina, San Diego, CA, USA). Each amplicon was sequenced using the MiSeq platform (Illumina) according to the manufacturer’s instructions.

### 2.3. Analysis of the Bacterial Composition of the Microbiome

Taxonomic assignment of the sequenced data was performed using the profiling program MDx-Pro ver. 1 (MD Healthcare, Seoul, Korea). After checking the read length (300 bp) and quality score (mean Phred score ≥ 20), high-quality reads were selected. Operational taxonomic units were clustered using the CD-HIT sequence clustering algorithm. UCLUST and QIIME were used for the taxonomic assignment. The bacterial composition was calculated at different levels. Genus-level cluster assignment was performed in the database. Histograms and heatmaps were prepared for the microbiome of patients with breast cancer and healthy controls. Alpha and beta diversity of microbiome assemblages were analyzed at the genus level. Estrobolomes from previous studies (Table 1) [10,12] were used for comparison.

### 2.4. Extraction of EVs Derived from S. aureus

*Staphylococcus aureus*, one of the bacteria listed in Table 1, was selected for the experiments. A strain of *S. aureus*, purchased from the American Type Culture Collection (ATCC 14458), and grown in Luria-Bertani broth at 37 °C and 200 rpm. A top-bottom vacuum filter (Corning, NY, USA) with a pore size of 0.45 μm and a QuixStand Benchtop System (GE Healthcare, Chicago, IL, USA) was used for filtration. Residual bacteria were removed from the supernatant using a bottle top vacuum filter with a pore size of 0.22 µm (Corning, NY, USA). The filtered extraction was ultracentrifuged at 150,000× *g* for 3 h at 4 °C on a type 45 Ti rotor (Beckman Instruments, Brea, CA, USA). After this step, the EV pellets were obtained and resuspended in PBS. The extracted EVs were stored at −80 °C [29].

### 2.5. Verification of the EVs Derived from S. aureus

The extraction of EVs was confirmed by transmission electron microscopy (TEM) and compared with previous data to confirm *S. aureus* EV [19]. Each EV solution was diluted using PBS, and 10 μL of the resulting suspension (50 μg/mL) and uranyl acetate (2%) were dropped onto a 300-mesh copper grid (EMS, Hatfield, PA, USA) for negative staining. A JEM1011 electron microscope (JEOL, Tokyo, Japan) was used for TEM. The dynamic light scattering distribution in 500 ng/mL EV solution was measured using a Zetasizer Nano ZS (Malvern Instruments, Worcestershire, UK) and the Dynamic V6 software.

### 2.6. Cell Viability Assay after EV and Tamoxifen Treatment

The MCF7 and BT474 cell lines (Korean Cell Line Bank, Seoul, Korea) were used to determine the effect of *S. aureus* EVs and tamoxifen in breast cancer cells. A total of 5 × 10^5^ cells were incubated in Dulbecco’s modified eagle medium supplemented with 10% fetal bovine serum. PBS was used to wash the cells, and fresh medium was added after 24 h of incubation. The EVs were then titered and diluted with distilled water. Cells in the control group were treated with distilled water, while those in the experimental group were treated with EVs at 10 ng/mL, 100 ng/mL, and 1 μg/mL concentrations for 72 h. *S. aureus* EVs (1 μg/mL) were used in co-treatment experiments with tamoxifen. Cells were treated with 1 μg/mL *S. aureus* EVs, 10 μM tamoxifen, or 10 μM tamoxifen plus 1 μg/mL *S. aureus* EVs for 72 h [31,32]. A trypan blue viability assay was performed to measure relative cell viability.

### 2.7. Western Blotting after Combination Treatment with S. aureus EVs and Tamoxifen

Cells were lysed, and equal amounts of proteins were separated by 10% sodium dodecyl sulphate-polyacrylamide gel electrophoresis (Bio-Rad Laboratories, Hercules, CA, USA). Protein bands were transferred onto Hybond™-ECL nitrocellulose membranes (Amersham Biosciences; GE Healthcare) to detect specific proteins. The membranes were then incubated overnight at 4 °C with anti-phospho-AKT1/2/3 rabbit polyclonal antibody (1:1000, Santa Cruz Biotechnology, Dallas, TX, USA), anti-phospho-ERK mouse monoclonal antibody (1:1000, Santa Cruz Biotechnology), anti-P21 mouse monoclonal antibody (1:1000, Santa Cruz Biotechnology), and anti-β-actin mouse monoclonal antibody (1:1000, Cell Signaling Technologies, Beverley, MA, USA). Labeled proteins were detected using a chemiluminescence detector (Amersham Bioscience; GE Healthcare).

### 2.8. Quantitative Real-Time PCR for Signaling Molecule Analysis

Treated cells were harvested with trypsin-ethylenediaminetetraacetic acid and washed three times with PBS. The cell pellet was treated with the RiboEx Kit (Ecocell, Hanam, Korea) for 5 min, after which chloroform was added for an additional 2 min; then, the mixture was finally centrifuged at 12,000× *g* at 4 °C for 15 min. Column chromatography was used for RNA extraction. cDNA was synthesized using the SuperScript III Kit for reverse transcription PCR, and quantitative real-time PCR (qRT-PCR) was performed using 2× Quantitect SYBR Green (QIAGEN) on LightCycler^®^ 96 SW 1.1 (Roche, Mannheim, Germany). Primer sequences for qRT-PCR are listed in Table 2, and they were the same as those used in a previous study [30].

### 2.9. Statistical Analyses

Student’s *t*-test and analysis of variance were used to determine the statistical significance of differences between groups. Microbiome data were averaged to select bacteria that accounted for more than 0.1% of the microbiota. Among them, only bacteria with a *p*-value of 0.01 or less were selected for analyses.

## 3. Results

### 3.1. Microbiome Analysis of Breast Cancer Patients and Healthy Controls

**Figure 1:**
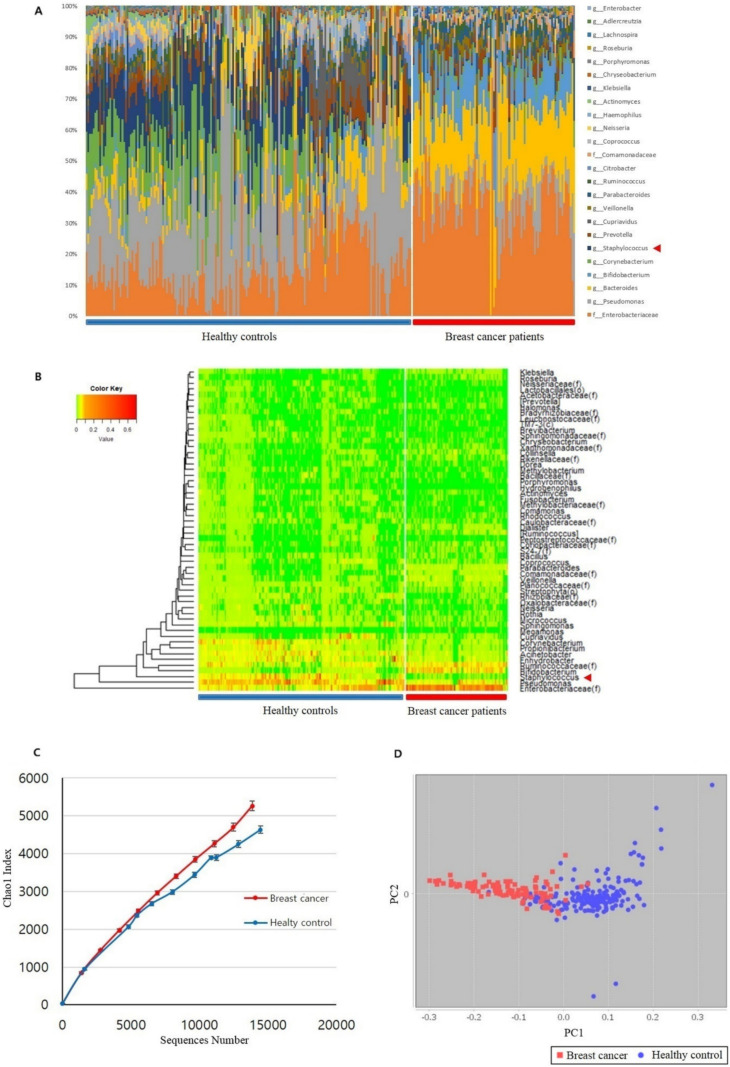

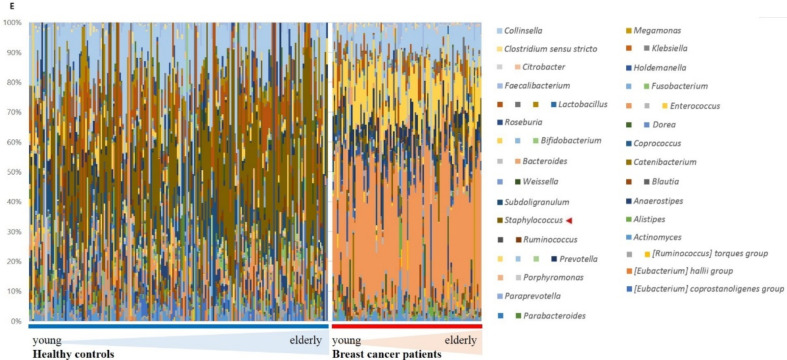
Microbiome analysis of blood samples from patients with breast cancer and healthy controls. (**A**) Microbiome differences between patients with breast cancer and healthy controls at the genus level indicated by a histogram. The red arrowhead indicates *Staphylococcus* sp. (**B**) Heatmap of microbiome differences between patients with breast cancer and healthy controls. The red arrowhead points to *Staphylococcus* sp. (**C**) Alpha diversity analyzed through the Chao1 index between the breast cancer and healthy control groups. The red and blue lines represent the breast cancer group and the healthy control group, respectively. (**D**) Beta diversity analyzed through the principal component coordinate analysis (PCoA) in patients with breast cancer and healthy controls. PC1, principal component 1; PC2, principal component 2; red dot, breast cancer patients; blue dot, healthy controls. (**E**) Association of the blood microbiome with estrogen metabolism. The bar chart depicts the genus−level percentages of the microbiome in healthy controls and breast cancer patients. Data were averaged to select for bacteria abundant in more than 0.1% of the microbiota. Bacteria listed in Table 1 were selected, and only those with a *p*−value of 0.01 or less were selected for analyses.

**Figure 2:**
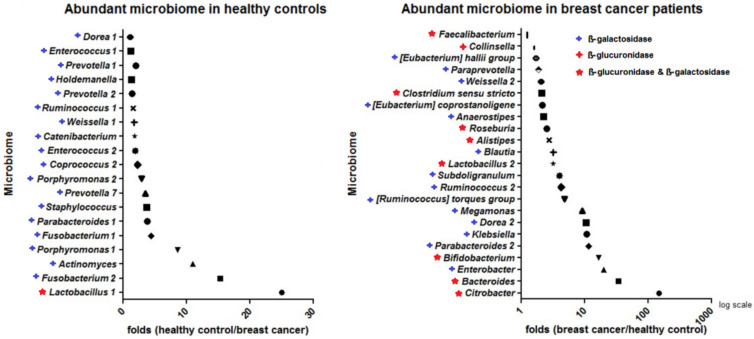
Comparison between blood microbiome composition of healthy controls and breast cancer patients. Three types of symbols indicate microbiota producing β−glucuronidase and/or β−galactosidase. As the data were acquired from sequencing at the genus level, when microbiota belonged to the same genus but to different species, the microbes with the same names were numbered differently.

The relative abundances of blood microbiome operational taxonomic units were determined in breast cancer patients and healthy controls (Figure 1). Blood microbiome differences between patients with breast cancer and healthy controls were demonstrated at the genus level using histograms and heatmaps (Figure 1A). The heatmap depicts significant differences between the breast cancer patients and the healthy control groups (Figure 1B). *Enterobacteriaceae*, *Bifidobacterium*, and *Ruminococcaceae* were abundant in patients with breast cancer, and *Pseudomonas* and *Staphylococcus* were abundant in healthy controls (Figure 1B). If the database is insufficient to assign a community at the genus level, these data are assigned at higher levels, such as *Enterobacteriaceae* and *Ruminococcaceae*. Alpha diversity demonstrates the diversity between the patients with breast cancer and healthy control groups. Beta diversity describes the diversity within each group of breast cancer patients and healthy controls. Alpha diversity was analyzed through the Chao1 index (Figure 1C). Beta diversity showed the differences via the PCoA plot between the breast cancer and healthy control groups by permutational multivariate analysis of variance (Figure 1D). Marked differences in diversity were found between the two groups (Figure 1C,D). Bacteria producing β-glucuronidase included *Collinsella* and *Edwardsiella*; bacteria producing β-galactosidase included *Dorea*, *Klebsiella*, and *Staphylococcus*; bacteria producing both the metabolites included *Alistipes*, *Bacteroides*, *Bifidobacterium*, *Faecalibacterium*, *Lactobacillus*, and *Roseburia* (Figure 1E, Table 1). *Staphylococcus* was more abundant in older healthy controls (over 40 years), but it was relatively absent in breast cancer patients (Figure 1E). *Bifidobacterium* was less abundant in healthy controls than in patients with breast cancer; however, its abundance among the breast cancer patients decreased with age (under 40 years) (Figure 1E). The predominant microbiome components in patients with breast cancer and healthy controls are shown in Figure 2. Bacteria that produce β-glucuronidase were predominant in the patients. In addition, *Citrobacter* and *Bacteriodes* were 149 and 34 times more abundant in the patients than in the healthy controls, respectively. *Enterobacter* and *Bifidobacterium* were at least 15 times more abundant in the patients with breast cancer than in the healthy controls. Furthermore, microbiota producing β-galactosidase were predominant in the healthy control group. For example, the abundance of *Lactobacillus* in healthy controls was 25-fold that in breast cancer patients and the abundance of *Fusobacterium*, *Porphyromonas*, and *Actinomyces* in healthy controls was at least 10-fold that in breast cancer patients.

### 3.2. Staphylococcus *sp.* Abundance in the Blood Samples

**Figure 3:**
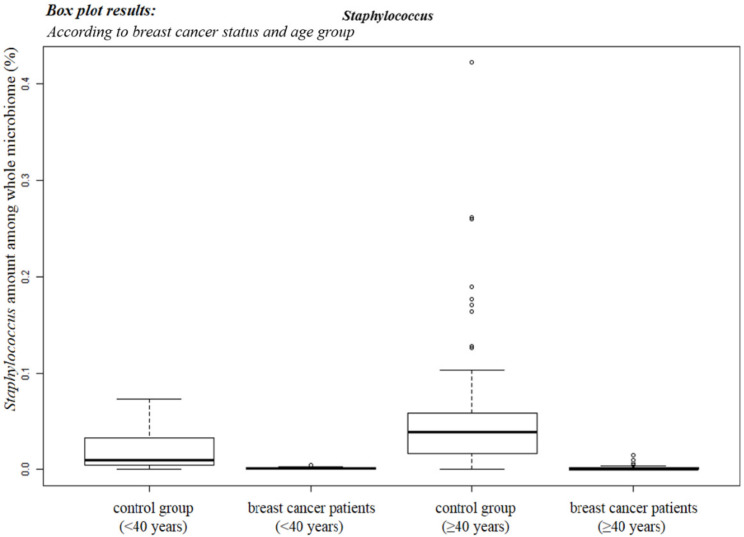
Average genus−level percentage of *Staphylococcus* in blood samples. The box plot shows the abundance ratio of *Staphylococcus* among the whole microbiota in both the healthy control group and breast cancer patients according to age. Error bars represent standard deviation.

The abundance of *Staphylococcus* sp. in the microbiota was determined by analyzing the blood of the 96 patients with breast cancer and 192 healthy controls. As the human microbiome changes according to age, data from young and old breast cancer patients (under and above 40 years of age, respectively) were analyzed separately (Figure 3). The abundance of *Staphylococcus* sp. was higher in the healthy control group over 40 years of age.

### 3.3. S. aureus EVs Confirmed by TEM

**Figure 4:**
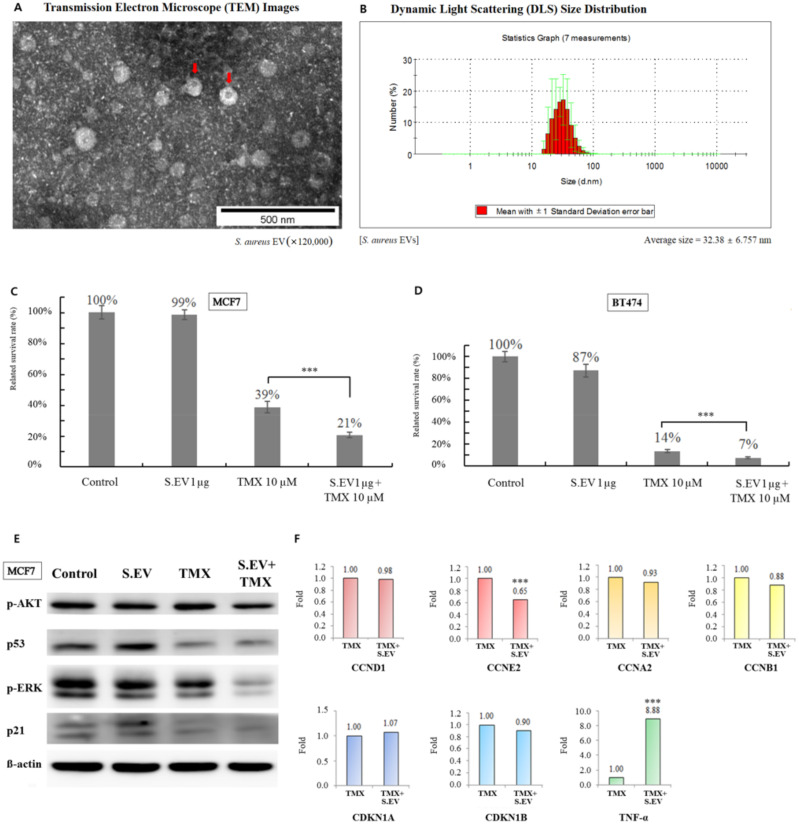
Effect of combination treatment with *S. aureus* EVs and tamoxifen in breast cancer cells. (**A**) EVs derived from *S. aureus* (indicated by a red arrow) were confirmed by transmission electron microscopy. (**B**) The average diameter of *S. aureus* EVs was obtained using dynamic light scattering size distribution. (**C**) The bar chart shows the percentage of viable MCF7 cells after treatment with *S. aureus* EVs and/or tamoxifen. (**D**) Relative BT474 cell survival percentages after treatment with *S. aureus* EVs and/or tamoxifen. This experiment was repeated three times. (**E**) Protein expression of *p*−AKT, p53, *p*−ERK, and p21 was detected by Western blotting after tamoxifen and/or *S. aureus* EVs treatment. lane 1, control; lane 2, *S. aureus* EVs 100 ng/mL; lane 3, 10 μM tamoxifen; lane 4, 10 μM tamoxifen plus *S. aureus* EVs 100 ng/mL. (**F**) The mRNA expression of cyclins, cyclin−dependent kinase inhibitors, and TNF−α was confirmed by qRT−PCR. CCND1, cyclin D1; CCNE2, cyclin E2; CCNA2, cyclin A2; CCNB1, cyclin B1, CDKN1A:p21, and CDKN1B:p27; TNF−α, tumor necrosis factor−α. *** *p* < 0.001.

The EVs extracted from *S. aureus* were confirmed by TEM (Figure 4A). Bacterial EVs derived from *Staphylococcus* are generally between 20 and 100 nm in diameter [33], and these *S. aureus* EVs were within this range. The average diameter of the *S. aureus* EVs in this study was 32.38 nm according to dynamic light scattering (DLS) size distribution (Figure 4B).

### 3.4. S. aureus EVs Enhanced Tamoxifen Efficacy for Breast Cancer Cells

Estrogen−receptor (ER) −positive MCF7 and BT474 breast cancer cells were cultured and treated with *S. aureus* EVs to observe their effect. ER−positive breast cancer cells treated with tamoxifen were set as the control group. Cells in the experimental group were treated with both *S. aureus* EVs and tamoxifen to monitor drug efficacy. Tamoxifen suppressed the percentage of viable cancer cells by 61−86 percent. The efficacy of tamoxifen was enhanced, and viable cancer cells were suppressed by 79−93 percent when combined with *S. aureus* EVs (Figure 4C,D). These results showed that these EVs improved the efficacy of tamoxifen on the growth inhibition of estrogen−receptor−positive breast cancer cells.

### 3.5. Signaling Molecules Involved in Increasing Tamoxifen Efficacy

The expression of *p*−AKT and *p*−ERK was downregulated upon combination treatment with tamoxifen and *S. aureus* EVs compared to treatment with tamoxifen alone in ER−positive breast cancer cells (Figure 4E). In addition, cyclin E2 was downregulated after the co−treatment. Conversely, TNF−α was found to be increased by greater than 8-fold after co−treatment with *S. aureus* EVs and tamoxifen compared to treatment with tamoxifen alone. Expression of p53 and p21 did not differ significantly between the tamoxifen alone and the co−treatment groups (Figure 4F).

## 4. Discussion

The microbiome is known to influence the pathogenesis of breast cancer [11]. Many studies have focused on the microbiome of patients with breast cancer and its effects [34]. The present study showed a new perspective of the microbiome in patients with breast cancer. Although many previous studies have been conducted on breast tissue, nipple aspirate, and stool samples [14,35], microbiome data of bacterial EVs in blood are rare.

Previous investigations found that β−glucuronidase−producing microbiota were abundant in breast cancer patients [14]. The present study focused on the microbiota producing β−glucuronidase and/or β−galactosidase. In the present study, the β−glucuronidase and β−galactosidase−-producing microbiota were 10 to 100 times more abundant in the cancer patients than in the healthy control group (Figure 2). The predominant bacteria in the breast cancer group were *Citrobacter*, *Bacteroides*, and *Bifidobacterium,* which are β−glucuronidase− and β−galactosidase−producing bacteria. These microbiotas might be involved in the development of breast cancer. Some microbiome components were more predominant in healthy controls, suggesting that they may play a role in preventing breast cancer and maintaining overall health. The predominant bacteria in the healthy control group were *Lactobacillus, Actinomyces*, *Fusobacterium*, *Parabacteroides*, *Porphyromonas*, *Staphylococcus*, and *Prevotella*. These results confirmed that the mechanisms involved in preventing breast cancer could be found in the abundant microbiota of the healthy controls. One of the predominant bacteria in healthy controls, *Staphylococcus*, was selected, and its EVs were used in combination with tamoxifen for treating breast cancer cells.

There are several reasons for choosing *S. aureus* for this experiment. First, the treatment of breast cancer involves estrogen receptors and endocrine therapy. *Staphylococcus aureus* is associated with estrobolome, which can affect breast cancer cells. Second, *S. aureus* is highly likely to have an impact on breast cancer treatment because metastasis prevention effects have been confirmed in breast cancer cells. Therefore, *Staphylococcus aureus* was selected from among the microorganisms that were abundant in the healthy control group and insufficient in the breast cancer patients. Among them, *S. aureus*, which is associated with breast cancer metastasis, was selected.

*Staphylococcus* is a bacterium that produces β−galactosidase [12]. It is abundant in elderly, healthy controls but almost absent in breast cancer patients (Figure 3). After estrogen treatment in a previous study, there was an interaction between the estrogen receptor (ER) and insulin−like growth factor (IGF) receptor [36]. Phosphorylation of ERK is required for the binding of ER to the IGF−1 receptor [37]. In addition, the PI3K and *p*−AKT pathways are mediated by membrane G protein−coupled estrogen receptors [38]. In the present study, *p*−ERK and *p*−AKT levels were increased when *S. aureus* EVs were used in combination with tamoxifen compared to tamoxifen treatment alone. Tamoxifen inhibits breast cancer cell growth by modulating PI3K/AKT, ERK, and IGF receptors [39]. Thus, *S. aureus* EVs affect tamoxifen efficacy by regulating *p*−ERK and *p*−AKT. Estrogen promotes cyclin D1 expression in breast cancer cells [40]. However, in the present study, cyclin D1 showed no statistically significant changes, suggesting that this process may not be related to the elevated tamoxifen efficacy. According to previous studies, elevation of cyclin E2 levels contributes to tamoxifen resistance [41], and decreasing cyclin E2 levels, as observed in the present study, are likely associated with an increase in tamoxifen efficacy.

A potential bias of the present study is that the microbiome was sequenced at the genus level. Therefore, there might be other species of *Staphylococcus* that could improve endocrine therapy efficacy in breast cancer cells. Certain types of *Enterobacteriaceae* are the most abundant microbiome in patients with breast cancer, but this cluster is not assigned at the genus level due to insufficient taxonomic information. Therefore, this is the second limitation. Third, as this study was conducted on blood samples alone, the microbiome might differ from that of other samples, such as breast tissues. However, to the best of our knowledge, there has been no previous research on this, making it a valuable foundation for further research.

## 5. Conclusions

The results obtained suggest that microbiome components can be applied to prevent and treat breast cancer. Additional animal and clinical trials will verify if *S. aureus* EVs can be used as adjuvants for breast cancer treatment.

## Figures and Tables

**Table 1:** Microbiota producing β-glucuronidase and/or β-galactosidase that were used for estrobolome analysis. The data were collected from previous studies on microbiome-related estrogen metabolism.

Genus	β-glucuronidase	β-galactosidase
*Actinomyces*	−	+
*Alistipes*	−	+
*Anaerostipes*	−	+
*Bacteroides*	−	+
*Bifidobacterium*	−	+
*Blautia*	−	+
*Catenibacterium*	−	+
*Citrobacter*	+	+
*Clostridium*	+	+
*Collinsella*	+	−
*Coprococcus*	−	+
*Dorea*	−	+
*Enterococcus*	−	+
*Eubacterium*	−	+
*Faecalibacterium*	+	+
*Fusobacterium*	−	+
*Holdemania*	−	+
*Klebsiella*	−	+
*Lactobacillus*	+	+
*Megamonas*	−	+
*Parabacteroides*	−	+
*Paraprevotella*	−	+
*Porphyromonas*	−	+
*Prevotella*	−	+
*Roseburia*	+	+
*Ruminococcus*	−	+
*Staphylococcus*	−	+
*Subdoligranulum*	−	+
*Weissella*	−	+

+: positive; −: negative.

**Table 2:** Primers used for qRT-PCR.

Gene	Type	Sequence (5′–3′)	MER	TM (°C)	GC (%)	Size (bp)
CCND1	Fw	CTCTGTGCCACAGATGTGAAG	21	57.9	52	170
	Rv	GAGGCAGTCCGGGTCACAC	19	57.2	68	
CCNE2	Fw	CTGGCTTTTAGAGGTATGTGAAG	23	56.0	43	162
	Rv	AGCATAGATTTCCTCAAGTTTGG	23	55.3	39	
CCNA2	Fw	GGACAAAGCTGGCCTGAATC	20	55.9	55	166
	Rv	GGAGAGAAACACCATGATACTTTG	24	55.2	42	
CCNB1	Fw	ATAATGGTGAATGGACACCAACTC	24	55.1	42	143
	Rv	ATACTTGTTCTTGACAGTCATGTG	24	54.8	38	
CDKN1A	Fw	ACCATGTGGACCTGTCACTG	20	54.8	55	135
	Rv	TGGAGTGGTAGAAATCTGTCATG	23	55.5	43	
CDKN1B	Fw	GACCTGCAACCGACGATTC	19	55.2	58	156
	Rv	TATTCTTAATTCGAGCTGTTTACG	24	55.3	33	
TNF-α	Fw	AGGCAGTCAGATCATCTTCTC	21	55.6	48	162
	Rv	CTGATGGCACCACCAGCTG	19	57.9	63	
GAPDH	Fw	GAGTCAACGGATTTGGTCG	19	57.5	53	133
	Rv	TGGAATCATATTGGAACATGTAAAC	25	57.8	32	

CCND1, cyclin D1; CCNE2, cyclin E2; CCNA2, cyclin A2; CCNB1, cyclin B1; CDKN1A, p21; CDKN1B, p27; TNF-α, tumor necrosis factor-α; GAPDH, glyceraldehyde 3-phosphate dehydrogenase; Fw, forward primer; Rv, reverse primer.

## Data Availability

Not applicable.
